# Reconsidering the Unwanted Sexual Touching of Boys by Adults: An Ethnographic Study in Rural Cambodia

**DOI:** 10.1177/08862605231153894

**Published:** 2023-02-20

**Authors:** Maurice Eisenbruch

**Affiliations:** 1 Monash University, Clayton, VIC, Australia

**Keywords:** cross-cultural attachment, child sexual abuse, cultural defense, genital touching, unwanted sexual touching

## Abstract

The unwanted sexual touching of boys by adults is a form of child sexual abuse. However, the genital touching of boys may be culturally “normal,” with not all instances necessarily being “unwanted” or “sexual.” This study, set in Cambodia, explored the genital touching of boys and the local cultural constructions of it. It entailed ethnography, participant observation, and case studies involving 60 parents, family members, caregivers, and neighbors (18 men, 42 women) in 7 rural provinces and Phnom Penh. Informants’ views, along with their use of language, proverbs, sayings, and folklore were recorded. The combination of the emotional driver for touching a boy’s genitals and the physical action of doing so is /krɨɨt/ (គ្រឺត or ក្រឺត). The motivation is usually overwhelming affection, and to socialize the boy to cover his nakedness in public. The spectrum of action ranges from light touching to grabbing and pulling. Benign and non-sexual intention is signaled by adding the Khmer predicative /tʰoammeaʔtaa/, or “normal,” as an adverb to the attributive verb /leiŋ/, or play. The genital touching of boys by parents and caregivers is not necessarily sexual in nature, though it is possible that abuse can be committed despite the absence of such intention. Cultural insights are not a “defense” or basis for exculpation, with each case evaluated simultaneously through cultural and rights-based lenses. There are anthropological implications in gender studies, and it is essential to have an understanding of the concept of /krɨɨt/ to ensure that interventions to protect the rights of children are culturally responsive.

## Introduction

Sexual touching has deep connotations in western traditions. Palmquist (2016) cited Proverbs 6:29 to show that, for the ancient Hebrews, the effect of touching was so strong that it was on par with sexual intercourse. Unwanted sexual touching (UST), is defined by UNICEF (n.d.) as “touching a person in a sexual way without their permission,” or “such as kissing, grabbing, pinching or fondling” and, according to UNICEF, is the most commonly reported form of child sexual abuse (CSA) (UNICEF, 2017). Studies such as the UN Multi-Country Study on Men and Violence in Asia and the Pacific (Fulu et al., 2013; UNICEF, 2012) have included UST as a marker of child sexual violence (CSV). CSA in Cambodia includes UST (Eisenbruch, 2019) and there do not appear to be any differences regarding definitions of UST in a global context versus a Cambodian context. UST can occur subtly or overtly in physically violent ways, especially in settings of conflict. UST can have long-term effects, including suicide attempts and non-suicidal self injury (Bikmazer et al., 2022) especially in the case of poly-victimization (Juan et al., 2019), posttraumatic stress disorder (Boumpa et al., 2022), and intimate partner violence (Miedema et al., 2023).

In the case of girls and young women, genital touching is invariably unwanted and sexual (Lyttleton, 2000; G. Miles et al., 2014; Stark et al., 2017). It is as serious for boys as well. Richter (2018) found that a third of the boys he surveyed in South Africa were subject to UST and/or sex. Is genital touching or grabbing of boys invariably unwanted and sexual or can it be “normal” in a cultural context? I was led to this question early in the Aṅgulimāla Walks research program on traditional healing in Cambodia, through my fieldwork observations, for example, where Cambodian men flick the penises of young boys as a sign of affection rather than in pursuit of sexual gratification (Eisenbruch, 1998, p. 511). If it can be shown that in Cambodia, it is “normal” to touch a boy’s genitals to express affection or for any other reason that is not “sexual,” is it necessarily UST and CSA? These are timely questions in a country where there is an imperative to create culturally responsive child protection interventions and education. I set out two sets of evidence, the first supporting the proposition that genital touching of boys is CSA, the second that it may not invariably be so.

## A Universalist Position

The *Clinical handbook: Health care for children subjected to violence of sexual abuse* (Ministry of Health, 2017a) defines unwanted sexual abuse as “touching a child in a sexual way, such as sexual kissing, touching a child’s genitals or private parts for sexual purposes, or making a child touch someone else’s genitals or play sexual games without their permission.” Thus, UST is CSA.

Genital touching is “unwanted” and “sexual” where boys are victims of CSA, exploitation, and trafficking. Hilton et al. (2008, pp. 96–97) described the experiences of boys who were abused by foreigners and adult Cambodians. In their baseline study on boys in Sihanoukville, Davis and Miles (2014) asked the respondents about being touched in the genitals since they were 8 years of age. This question aimed to distinguish sexual touching from the common cultural practice in which adults touch or kiss the genitalia of young men. A third of the boys said that they had been sexually touched, usually from the age of 12 years onward. Davis et al. (2017, p. 27) investigated the experiences of street children on the Thai–Cambodian border, and reported that nearly one-fourth had said that an adult had touched them inappropriately in the genital area. The UST of boys has also been described in temples, where Buddhist monks have manipulated the penises of novice monks to the point of ejaculation (Tarr, 1996, p. 104). It is important to learn more about the cultural landscape behind the UST of these highly vulnerable boys. The common practice of touching a child’s penis by an adult does not necessarily make it “normal.” In G. M. Miles (2016) and Hilton et al. (2008) boys reported having been sexually touched in the genitals by adults since they turned 9 years of age. More boys (18.9%) than girls (13.5%) had reported this. Hilton et al. (2008) noted that the reason for such practices was reported as teasing and/or joking.

## Anthropological Challenges to a Universalistic Approach to the Rights of the Child

The anthropological literature suggests that genital touching of boys is not invariably CSA. Korbin (1987, p. 250) wrote that “. . . proscribed sexual conduct with children is likely to occur in extreme secrecy. If small children’s genitals are fondled openly, with other adults present, this must be considered differently than fondling of children that takes place secretly.” She also noted that where children’s genitals were fondled to please them, “This would not constitute ‘abuse’ if in that society the behavior was not proscribed and was not for the purposes of adult sexual satisfaction.” Evolutionary researcher Dagg (2014) felt impelled to look for the roots of “molestation” in evolutionary theory and argued that human ancestors shared a tendency to fondle the genitalia of their young as primates, as an expression of bonding and attachment that makes evolutionary sense. Could it also be true that human behavior is not very different, and that genital touching is not sexual?

Ford and Beach (1951, p. 112) described how parents in some societies fondled the genitals of their young children in a manner that is not considered “abusive” (see Mead’s (1952) critique of the authors’ anthropology). The best-known classic examples were provided by Mead (1935), who wrote about nursing Arapesh mothers slapping their children’s genitals playfully (p. 41); DeMause (2009), who wrote about societies in which mothers masturbate their young sons and suck their penises; and Malinowski (1929), who watched the widespread sucking of children’s genitals as a practice encouraged by parents. Fischer et al. (1976, p. 203), noted that Ponapeans acknowledge the attractiveness of children’s genitals, referring to the “widespread custom of playing with a baby affectionately by lifting it up and sniffing or blowing on the genitals.” Olson (1981), cited in Korbin (1987, p. 255), reported that adults in Turkey kiss and praise the genitals of young children, celebrating their eventual fertility. Josephs (2011, p. 14), citing Holmes (2007) on the importance of touch in the caregiver/infant relationship in strengthening attachment, addressed the “considerable cross-cultural variability in what is considered interpersonally sensitive touching of children’s genitals” and suggested that this variability could be “a relatively recent product of the cultural evolution of sexually conservative traditions. . .. rather than an adaptive species-wide norm that evolved long ago in the original environment of evolutionary adaptedness” (p. 14). Children may be unconsciously perceived by parents to possess “cute, adorable” genitals, which activates desires in them that can provoke disgust among outsiders.

The non-sexual genital touching of boys is reported widely in Asia. Michaelson (2004) reported that in Vietnam, adult relatives fondling their baby boys is an innocuous way of showing affection. Shirokogoroff (1973) reported that mothers from the Manchu community suck their sons’ penises as a means of soothing and strengthening their attachment (Halperin et al., 1990). Malley-Morrison and Denise (2004, p. 195) described how half the Vietnamese and Korean mothers, and 28% of Cambodian mothers found it acceptable for a grandfather to proudly touch the genitals of his grandson aged 3 years. Villarta-De Dios (2022) described how Filipino parents perceived holding and kissing genitalia (*humawak o humalik sa ari*) as appropriate in the context of hygiene and affection.

Cambodia offers a fertile ground for research on genital touching among boys. Even today, there is nothing remarkable if, on occasion, a man grabs another man’s genitals, even on national television (Ream Production, 2021). This article addresses the common practice, at least in rural Cambodia, of adults showing affection to young boys by touching or kissing their genitals (Yaim, 2018). The literature shows that the motives are far from those that inform sexual abuse, for example, to soothe boys up to the age of 3 years (Blanch et al., 2012, pp. 410–411; G. Miles and Sun, 2006); as a joke in the case of boys aged between 2 and 4 years (Hilton et al., 2008); and to check for the presence of a penis in the case of boys with feminine facial features (G. Miles and Sun, 2006, p. 36). Blanch and Miles (2012, p. 411) stated that, “Culturally, these are not considered as abusive actions and there are even debates in discussions among those involved in child rights regarding whether it is abusive.”

This paper explores the hypothesis that rural Cambodians consider the touching of the genitals of young boys a normal expression of love or care rather than sex, and that if anything the possible dangers are of physical damage rather than sexual abuse. It documents the practice of genital grabbing of boys by adults to explore the local cultural construction to test the proposition that touching a boy’s genitals is normative, rather than CSA in Cambodia. First, I present the findings on the manner in which adults express their feelings of affection, admiration, and care for a boy by the physical action of reaching out and handling his genitals. I delineate the motivations, most commonly as the affection of the adult bursting forth, but sometimes as a means of socializing boys, to teach them through shame and discomfort to cover their genitals when in public. I describe this combination of the emotional driver and physical action as the Cambodian concept of /krɨɨt/ (គ្រឺត or ក្រឺត). I then describe the spectrum of actions taken, ranging from innocuous and incidental light touching all the way to rough manipulation grabbing and pulling and even inserting the penis into the mouth. Then I show how the benign and non-sexual intention is signaled by adding the Khmer predicative /tʰoammeaʔtaa/, or “normal,” as an adverb to the attributive verb /leiŋ/, or play. I discuss the cultural context for the genital touching of boys, showing that these actions should not automatically be considered UST, and raising cautions, however, that cultural practices should never become a blind spot and that some boys could be sexually abused, even if the adult does not realize it at the time. I discuss the anthropological implications in gender studies and flag the importance of understanding the concept of /krɨɨt/ in ensuring that interventions to protect the rights of the child are culturally responsive.

## Method

The research questions were framed in consultation with community members, including Buddhist monks and those who had lived experience of interpersonal violence (Tajima, 2021). Care was taken not to lump together the concerns of diverse groups within Cambodian society (Bent-Goodley, 2021). It is common knowledge that, whereas the touching of a girl’s genitals under any circumstances (other than for hygiene or medical purposes) is universally considered CSA, that of boys does not seem to be considered thus. As we continued to focus on CSA among the Buddhist temple community from 2017 to 2021, we saw that some monks and ritual officiants sexually exploited the temple boys and novice monks.^1^

### Sampling

The sample comprised 60 informants (18 men, 42 women) who were typical in terms of their religious, linguistic, cultural, and social diversity as Buddhist rice-farming villagers (Table 1). These comprised 27 family members (11 mothers, 1 father, 10 grandmothers, 4 grandfathers, 1 aunt); 162 villagers (13 women, 3 men); and 17 key informants comprising 7 village heads (2 women, 5 men), 4 female nannies, and 6 people from the religious sector (1 female medium, 4 ritual officiants, 1 monk).

**Table 1.** Demographic Characteristics of the Informants.

ID	Age	Sex	Status of Informant	Commune	District	Province	Rural/Semi-rural
CC	60	M	Village head	Phsar Daek	Ponhea Lueu	Kandal	R
CH	60	F	Villager	Baray	Kampong Leav	Prey Veng	R
CN	70	F	Grandmother	Khmuonh	Sen Sok	Phnom Penh	R
CS	66	F	Grandmother	Phsar Daek	Ponhea Lueu	Kandal	R
CSn	66	F	Village head	Phsar Daek	Ponhea Lueu	Kandal	R
DD	65	M	Villager	Chhveang	Ponhea Lueu	Kandal	S
EH	66	M	Grandfather	Roung Damrei	Kampong Leav	Prey Veng	R
HB	48	M	Village head	Tuol Sangkae	Ruessei Kaev	Phnom Penh	S
HH	64	M	Village head	Chhveang	Ponhea Lueu	Kandal	S
IC	76	M	Villager	Trapeang Kong	Samraong Tong	Kampong Speu	R
IP	73	M	Grandfather	Svay	Samaki Meanchey	Kampong Chhnang	R
ISL	54	F	Nanny	Phsar Daek	Ponhea Lueu	Kandal	R
KCC	21	F	Mother	Phsar Daek	Ponhea Lueu	Kandal	R
KK	70	F	Nanny	Khmuonh	Sen Sok	Phnom Penh	R
KL	79	M	Acaa	Chrey Loas	Ponhea Lueu	Kandal	R
KLai	49	F	Villager	Phsar Daek	Ponhea Lueu	Kandal	R
KN	81	M	Acaa	Chrey Loas	Ponhea Lueu	Kandal	R
KNal	58	F	Mother	Khmuonh	Sen Sok	Phnom Penh	R
KP	46	M	Father	Svay	Samaki Meanchey	Kampong Chhnang	R
KS	33	F	Mother	Champa	Prey Kabbas	Takeo	R
KSO	33	F	Villager	Chhveang	Ponhea Lueu	Kandal	S
KT	28	F	Mother	Khmuonh	Sen Sok	Phnom Penh	R
LD	31	F	Villager	Samraong Tboung	Samraong	Phnom Penh	R
LR	31	F	Mother	Me Pring	Batheay	Kampong Cham	R
MS	81	M	Acaa	Preah Bat	Steung Treng	Stung Treng	R
MY	70	F	Villager	Kantueu Pir	Banan	Battambang	R
NC	52	F	Mother	Phsar Daek	Ponhea Lueu	Kandal	R
NChe	73	M	Grandfather	Chrey Loas	Ponhea Lueu	Kandal	R
NK	58	F	Mother	Svay	Samaki Meanchey	Kampong Chhnang	R
NL	68	F	Grandmother	Chrey Loas	Ponhea Lueu	Kandal	R
NS	67	F	Villager	Kaoh Chen	Ponhea Lueu	Kandal	R
NS	30	F	Villager	Chrey Loas	Ponhea Lueu	Kandal	R
PC	37	F	Mother	Kaoh Chen	Ponhea Lueu	Kandal	R
PES	72	F	Medium	Baray	Kampong Leav	Prey Veng	R
PP	64	M	Village head	Tumnob Thum	Ponhea Lueu	Kandal	R
PS	63	M	Villager	Nimit	Poipet	Banteay Meanchey	R
PSok	68	M	Monk	Pot Sar	Bati	Takeo	R
PSy	65	F	Villager	Me Pring	Batheay	Kampong Cham	R
PY	36	F	Villager	Baray	Kampong Leav	Prey Veng	R
RS	64	F	Villager	Phnum Bat	Ponhea Lueu	Kandal	R
RSa	73	F	Grandmother	Khmuonh	Sen Sok	Phnom Penh	R
SC	63	F	Grandmother	Khmuonh	Sen Sok	Phnom Penh	R
SN	50	F	Villager	Svay	Samaki Meanchey	Kampong Chhnang	R
SNa	35	F	Mother	Spean Thma	Dangkao	Phnom Penh	R
SNa	55	F	Grandmother	Me Pring	Batheay	Kampong Cham	R
SP	65	F	Villager	Me Pring	Batheay	Kampong Cham	R
SV	52	F	Village head	Me Pring	Batheay	Kampong Cham	R
TM	52	F	Grandmother	Spean Thma	Dangkao	Phnom Penh	R
TSN	29	F	Villager	Spean Thma	Dangkao	Phnom Penh	R
TT	55	M	Village head	Veang Chas	Oudong	Kampong Speu	R
US	55	F	Nanny	Phsar Daek	Ponhea Lueu	Kandal	R
UY	77	F	Grandmother	Khmuonh	Sen Sok	Phnom Penh	R
VN	70	F	Grandmother	Svay	Samaki Meanchey	Kampong Chhnang	R
VP	84	M	Acaa	Kantueu Pir	Banan	Battambang	R
VS	80	F	Mother	Kantueu Pir	Banan	Battambang	R
XA	25	F	Aunt	Boeng Kak	Tuol Kouk	Phnom Penh	M
XB	60	F	Grandmother	Boeng Kak	Tuol Kouk	Phnom Penh	M
XC	65	M	Grandfather	Boeng Kak	Tuol Kouk	Phnom Penh	M
YH	42	F	Mother	Roluos	Dangkao	Phnom Penh	R
YN	72	F	Nanny	Baray	Kampong Leav	Prey Veng	R

In many families today, parents are away from home to earn a living, and leave their children with their grandparents as caregivers. Grandparents are a strategic source of information about genital touching.

### Procedure

As part of our Aṅgulimāla Walks program, we obtained ethics approval from the National Ethics Committee for Health Research in Cambodia. We used our existing networks of village leaders, Buddhist monks, and lay ritual officiants to identify suitable families. The Sexual Violence Research Initiative’s Ethical Guidelines state that oral consent is preferable (Jewkes et al., 2012). Based on the Participant Explanatory Statement and our nuanced prompts, we ensured that our research would not be misconstrued as condoning CSA as a cultural or religious aspect of Cambodian life. Informants’ names and locations have been anonymized. The fieldwork encounters were carried out between 2014 and 2022 by the author, a male medical anthropologist and transcultural psychiatrist based in Australia, and two Cambodian assistants (Mr CA and Ms CP). In terms of researcher positionality (Tajima, 2021), the author writes as a middle-class man with an acquired understanding of Cambodian Buddhism and the Khmer language, but with sufficient cultural humility to appreciate the expression, “Khmer speech, foreign heart.” Mr CA is a male native Cambodian with over 30 years’ experience of having been trained by the author in the ethnographic method and carrying out fieldwork with him in urban and rural areas. He has longstanding relations with many of the informants. Ms CP is a female native Cambodian with a background in Khmer linguistics who had worked with the author for 12 years both in Australia and Cambodia at the time of writing. Other female and male research assistants were recruited from time to time. Wherever possible, women interviewed women, and men interviewed men. However, this became difficult between 2020 and late 2022, owing to COVID-19-related restrictions, following which the author and Ms CP traveled from Australia to Cambodia.

We documented the informants’ views on the genital touching of boys and recorded their use of language, proverbs, sayings, and their views on relevant folklore. We explored the ways in which communities made sense of the genital touching of boys, and the ways in which the informants understood the terminology around UST that is used as part of CSA programming, notably with the Khmer phrase /kaa pah poal/ + /pləv pʰeet/ + /dael kee mɨn pratnaa/ (ការប៉ះពាល់ផ្លូវភេទដែលគេមិនប្រាថ្នា). We also explored the construct of “abuse” /bɑmpien/ (បំពាន) and sexual abuse, /rumloup bɑmpien/ (រំេលាភបំពាន). In noting the borderlands among innocuous and sexual touching, and physical manipulation, we recorded the vernacular language, /pah poal/, conveying the sense that a light touch, far from being random, was carried out with intent, either positive as in caring and expressing love for the boy or, in causing harm. The expressions depicting the motives for action included overenthusiastic play (“play that went too far, a slip of the hand,” /leiŋ crul day/ *n* = 9); an intention to inflict pain or harm (“having an awful intention” /bɑmnɑɑŋ ʔaakrɑk/ (បំណង អារកក់); *n* = 4), and the use of rough action /bɑmpien/ (*n* = 2), and anger, /kʰəŋ/ (ខឹង).

Rather than applying the developmental stage of the child as commonly defined (Day et al., 2013; Ministry of Health, 2017b) using western criteria, we used the popular groupings used by the informants to depict a boy’s development. A child of any age is a young person who is under the control of someone else, a /kmeɛŋ/ (េមង) (*n* = 9). Those aged between birth approximately 4 or 5 months are called by the onomatopoeic sound of a baby crying, /koon ŋaa/ (កូនង៉ា) or /koon ŋaet/ (កូនង៉ែត). Children aged between 1 and 3 years are “small small children, /kmeɛŋ tooc tooc/ (េកងតូចៗ) (*n* = 24) and those aged between 3 and 7 years are “small children,” /kmeɛŋ tooc/ (េកងតូច) (*n* = 21). Children aged between 8 and 11 years are “big children,” /kmeɛŋ tʰom/ (េកងតំ) (*n* = 5) and those aged approximately 12 years and above are “big big children” /kmeɛŋ tʰom tʰom/ (េកងធំំធំ). Ethnographic encounters offer high data saturation as it takes long to gather multiple in-depth encounters and many methods are used (Fusch et al., 2015). Fieldwork continued until there was enough information to replicate the study.

### Analysis

The author and CP used thematic analysis to seek the insider views of people within the culture, narrative inquiry of the storied lives of particular cases, and grounded theory (Bhattacharya, 2017; Levitt et al., 2018; Pham et al., 2021). The starting point was a “theoretical sensitivity” (Thistoll et al., 2016) acquired through years of fieldwork experience with CSA in Cambodia. We started by identifying the ways in which genital touching took place, for example, /cʰək/, and the feelings that seemingly drove the action, for example, /krɨɨt/, as it emerged during fieldwork. We analyzed the metaphors conveyed by the informants, for example, the depiction of the sexual maturation of a boy in terms of types of fish. It was essential to grasp how the informants construed the boundary between “normal” and “violation,” and we analyzed their use of language, for example, how they added the Khmer predicative /tʰoammeaʔtaa/, or “normal,” as an adverb to the attributive verb /leiŋ/, or play. We documented how the informants attributed a compound verb /pah poal/ (ប៉ះពាល់), “touch and manipulate,” as a marker of physical or sexual abuse or as neutral, based on the sex of the child. We chunked these data, pulling out phrases and exploring the patterns. We identified the cultural idioms for genital touching, noting the cultural registers and use of popular Khmer cultural references. Care was taken to achieve cultural rather than merely linguistic equivalence (Tajima, 2021). We coded the action carried out by the toucher (e.g., /cʰək/), first, by grouping the cluster of actions described by each informant, then by extracting the most extreme level of action described and, finally, by indexing the case in which the action was described as excessive. We coded the emotional drivers of filial love and affection (e.g., /krɨɨt/), then those where there was some malevolence, and finally those where /krɨɨt/ was stated as the hybrid of action expressed as the ultimate expression of filial love. The motive was coded as filial affection (A) or socialization (S) such as, for example, to avoid nakedness in public. The opinion on whether touching was considered “normal” or “abusive” was coded: normal in any context (N) or in the family circle (F); and “abusive,” either as “sexual abuse” (S) or “*potentially* physical abuse” if there were concerns that overly rough handling to lead to physical injury of the genitals (C). We emphasize the use of Khmer terms as local concepts and idioms cannot be translated precisely into English. We avoid the “category fallacy” that emanates from simply translating words into English. Khmer terms are spelled using Huffman, Lambert, and Im’s (1970) adaptation of the International Phonetic Alphabet transcription rather than transliteration, to help non-speakers pronounce the terms easily and consistently. Words and expressions in Khmer characters are included.

## Results

An adult in a position of authority, for example, a parent, grandparent, or another senior relative, or a neighbor who is familiar with the family, may /krɨɨt/ a boy. A father may reach out to his son when he sees him naked. Caregivers may do so during intimate moments such as while bathing children. Touching the penis is most common in the first 5 years of life, and can continue into adolescence. The informants used the general term “youngster, youngster,” /kmeɛŋ kmeɛŋ/ (ក្មេងក្មេង) to refer to children. The most “natural” person to touch or grab a boy’s genitals other than for the purpose of hygiene in was his father. Relatives can include grandparents, uncles, aunts, and cousins. We had known PS for some years in the course of fieldwork, and he said that a male relative tends to touch or grab the penis directly, whereas a female one tends to pat the boy’s bottom, and close friends and neighbors may also grab the penis. It is considered inappropriate for monks and strangers to do more than pat a boy’s bottom.

### Emotional Drivers

#### Cascading Attraction and Acting on /krɨɨt/

Parents, family members, and caregivers are often motivated by attraction toward young boys, which manifests in the form of touching their genitals. The starting point is the feeling of appreciation, affection, and love, known in Khmer as **/**srɑlaɲ/ (ស្រឡាញ់), and does not convey sexual love. As many as 54 of the 60 informants indicated affection as the motive for genital touching. When used in the serial verb **/**srɑlaɲ roap? aan/ (ស្រឡាញ់រាប់អាន), **/**srɑlaɲ/ means friendship rather than parental love. As many as 10 of the 60 informants indicated **/**srɑlaɲ roap? aan/ as a motive for genital touching. With the intensification of these feelings, the adult becomes entangled in their attraction toward the boy, a state of “loving a lot, almost too much,” /knaɲ/ (ក្នាញ់ or ខ្នាញ់). SC explained how she wanted to devour her grandson, and it was only when she reached out to kiss him from top to toe including his genitals that she was relieved of her “hunger.”

When it intensifies further, the compounding of the strong feeling and the resultant action is known as /krɨɨt/. This overflow of **/**knaɲ/ into /krɨɨt/ is known as the predicative /krɨɨt knaɲ/. The **/**knaɲ/ cannot be contained and the parent or caregiver cannot resist fondling the boy’s genitals. Most people would be familiar with the language of /krɨɨt/ and its intensifier /krɨɨt knaɲ/, and would be aware that the terms are defined contextually, as village head CSn specified: the feelings and actions of lovers or married couples in the intimacy of their bed clearly denotes sexual arousal, as expressed through the feeling of “inflaming the heat of desire” /rumcəəp - rumcuəl/ (រំជើបរំជួល). CSn said that, in contrast, there is none of that when a parent touches, fondles, or cradles their son’s genitals as these are expressions of affection without a trace of sex or of intention to “reach the point.” As CSn said, people speak of /krɨɨt knaɲ/ of the genitals as something done to boys, never to girls. Several informants said that they “loved [the boy] almost to the point of wanting to swallow [him],” /srɑlaɲ stǝǝ leep/ (ស្រឡាញ់ស្ទើរលេប), and that they reached out as if to eat the delicious boy and, in the process, touched his genitals. Older informants expressed this through the metaphor of the legend of Rahu. UY said that she drew the line at touching the boy’s penis, unlike other women who said they may go as far as to suck it. This sense of the danger of overexuberant expression of love to the child was expressed by five older women and men, mostly grandparents—EH, SC, NK, RS, and UY (aged 66, 63, 70, 73, and 77 years, respectively) who remembered folktales from their elders that spoke of the legend of Rahu, a figure in ancient Hindu mythology. Overwhelmed by nostalgia and love for his siblings, the sun and moon, Rahu swallowed the former by day during a solar eclipse and the latter by night during a lunar eclipse, although he meant no harm and quickly spat them out. EH had moved to Phnom Penh from his home town in Svay Rieng, leaving behind most of his 11 grandchildren and during the long intervals his /srɑlaɲ stǝǝ leep/ accumulated until they were reunited, when he just had to appreciate his grandsons’ penises. He picked them up, headbutted them, and fondled their penises—but, like Rahu, only for a short time—and then released them. EH saw a simile between his and Rahu’s actions.

SC was a grandmother from Kampong Speu, who wanted to /krɨɨt/ the smallest and plumpest of her grandchildren. Her grandparents had taught her the ancient folk tale of Rahu, and she talked about the attraction of Rahu for the planetary bodies, especially for her favored and littlest one, the Moon. UY echoed SC’s view that adult-sized Rahu would firmly embrace the child-sized Moon and swallow him slowly, and regurgitate him or have him emerge through his ribcage, which was a good omen. However, if Rahu “went the whole hog” by retaining him, the Moon would die. SC joked that were it not for his ears sticking out, she would have gladly swallowed her grandson whole, but said that going any further would be like an “adult-sized” snake swallowing a “child-sized frog.”

#### Socialization

As boys grew older, they had to learn to cover up their genitals. To socialize them, adults and parents tended to reach out and grab the exposed genitals to inflict discomfort and sometimes to shame them into covering their genitals. A total of 31 of the 60 informants indicated socialization as the motive for genital touching. Some parents would chase after their sons, threatening castration using the cattle farming expression, “geld,” /kriəv/ (គ្រៀវ) and shouting, “Cover up your private parts or I’ll cut off your balls!” PES, 72 years, was a spirit medium in Prey Veng and was well-respected for her ability to connect with the notable guardian spirts of the region. YN boarded in her house, and they were involved in caring for various young children. The two told us they disciplined the older boys under them through threats while chasing them: “Go, boy, put on your pants or I’ll geld you,” /kriəv/ (គ្រៀវ). Even more vicious expressions used were, “Cover up, or I’ll chop it off,” /kat caol/ (កាត់ចោល), and “It’s dangling! Watch out! A dog will grab your genitals and run off with them,” /rɔyiiŋ-rɔyooŋ, prɑyat, ckae poam bat/ (រយីងរយោង ប្រយ័ត្នឆ្កែពាំបាត់). Horrible as these expressions sound, the intention is to socialize the boys for their own good.

#### Nuancing the Maturation of the Boy Whose Genitals Are Touched

Rural people conveyed the sexualized image of a boy on the way to maturation through animal metaphors such as a fish to denote the penis, in which /krɨɨt/ takes shape. RS, an elderly fisherwoman who lived in the fishing area of the Prek Pnov waterway, graded the pike. The smallest was /trəy diep/ (ត្រីដៀប), followed by a larger species called /trəy cdao/ (ត្រីឆ្ដោ), and the plumpest, biggest, and delectable species, called /  trəy krɑmal/ (ត្រីក្រមាល់) or /trəy kʊəl reaŋ/ (ត្រីគល់រាំង). RS knew this taxonomy, but used a slightly different logic based on /krɨɨt/. She likened the youngest boys to the /  trəy krɑmal/ because, with their puppy fat, they were irresistibly and mouth-wateringly soft and chubby, as CSn said, /trɔluk trɔlʊən/ (ទ្រលុកទ្រលន់) and as PS called them /mien sac krɑmal mɑl/ (មានសាច់ក្រមាល់មាល់), also known as a /trəy kʊəl reaŋ/ (ត្រីគល់រាំង). The next is the “undersized pike” a /trəy diep/ (ត្រីដៀប), which fits in with the description NK and KP offered for their disabled son who had undeveloped genitals. Following this, is the pubertal boy, who is thriving and whose genital bundle is developing rapidly, and a few of those who were immature could neglect to cover themselves. RS called these /sraat cdao/ (ស្រាតឆ្ដោ), or literally, “naked pike fish.” She said that older men and women felt impelled, without any sexual excitement, to reach out to grab such boys’ genitals.

### Spectrum or Gradient of Action

There was a gradient of touching based on the degree of physical discomfort or pain inflicted. The mildest level was depicted as /pah/ (ប៉ះ), a simple touch that may have been accidental, with deliberate contact with the boy’s penis, /poal/ (ពាល់) or just a light caress, /stiep/ (ស្ទាប). If the adult feigns the movement but deflects at the last moment, it is called /cʊənlɔɔ/ (ជន្ល). If the adult reaches out to gently cradle the genitals in their hand, /trɔɔ / (ទ្រ) and bounces them lightly, like a tennis player about to serve, it is called /treh/ (ត្រែះ). There is no infliction of pain. The second level involves the infliction of physical discomfort. In the verb /cʰək/ (ឆឹក), the adult rubs the penis with a finger. In the verb /ckəh/ (ឆ្កឹះ), the person scratches the penis a bit. In the serial verb /cap cbəc/, the adult grabs /cap/ (ចាប់) the boy to restrain him and then, in the manner of a fish /cbəc/ (ច្បិច) or a crab nipping, /kiep/ (កៀប), a bee stinging /kdec/ (ក្ដិច), a cat scratching with all its claws /krɑɲav/ (ក្រញៅ) or a dog biting /kʰam/ (ខាំ), pinching or twisting the foreskin or scrotum between the fingernails. Some people reach out with the index finger to flick the penis so that it jerks, as if erect, /ptoat/ (ផ្ទាត់). In the third level, pain is inflicted. In /cboot/ (ច្បូត), /tieɲ/ (ទាញ), /crɑbac/ (ច្របាច់), /muəl/ (មួល) and /bok/, the adult grasps the penis by the hand and presses, yanks, strongly massages, twists, and pounds it so that it hurts.^2^Table 2 shows the frequency of actions taken.

**Table 2.** Frequency of Actions Taken in “Touching.”

/cʰək/	36
/cbəc/	25
/pah/	16
/kdəc/	15
/treh/	11
/tʰaəp/	11
/pah poal/	10
/bəc/	10
/krɨɨt/	9
/poal/	5
/biem/	4
/kiep/	4
/ptoat/	3
/cap/	3
/trɔɔ/	3
/cʊəɲcʊək/	2
/cʰək cʰək/	2
/bɑɲcʰək/	2
/tieɲ/	2
/stiep/	2
/ɲʊəl/	1
/muəl/	1
/biəm/	1
/cul/	1
/bok/	1
/pracʰək/	1
/kʰam/	1
/bəət/	1
/cak/	1
/bav/	1
/ʔɑŋʔael/	1
/prɑcʰək/	1
/luuk/	1
/cboot/	1

To grade the severity of the action, one must understand the underlying motive. In the second level, some informants indicated that their intention was to check whether the penis was in good condition and could become erect. NChe freely /treh/ naked boys in the village and, even though the touching gave a boy an erection, the parents laughed and treated it as good-natured fun. NChe had to be careful not to arouse the ire of these boys’ parents, especially when he accidentally traumatized the boys’ genitals, as it would make them angry with him and cause them to lose face in the eye of the public, which would consider them deficient parents. In the third level, where a pre-pubertal boy ran around naked, the person’s motive was to socialize him by hurting his penis a little, for example, with the intention of shaming him into wearing pants. There is a blind spot where boys can be sexually abused through genital touching. For girls, the compound verb /pah poal/ (ប៉ះពាល់) always had a bad implication of sexual abuse. For boys, /pah poal/ does not connote sexual abuse.

#### Oral Contact

A few informants described an action performed using the mouth rather than the hand. In a single action, a person kisses /tʰaəp/ (ថើប) the penis, puts it into their mouth, /biem/ (បៀម), sucks and slurps on it /bəət/ (បឺត), and give it a bit of a nip /kʰam/ (ខាំ). In a continuous action, the person sucks on the penis as if feeding on the breast /bav/ (បៅ) and, with greater enthusiasm, they suck like a leech drawing blood until it is satiated /cʊəɲcʊək/ (ជញ្ជក់). EH, an elderly rice farmer from a remote village in rural Prey Veng province, moved to Phnom Penh, leaving his extended family behind. When they visited him in Phnom Penh, he excitedly kissed his grandson, headbutted him, caressed his buttocks, and lovingly caressed and pinched his genitals, saying, “Gosh, dear grandson, your balls and your prick may be little, but they are absolutely perfect!” /aa cav nih tuəc lʔɑɑ nah vəɨy/ (អាចៅនេះតួចល្អណាស់វើយ!). In doing so, he reaffirmed the intergenerational bond /puuc/ (ពូជ) between grandfather and grandson.

KK, aged 70 years, lived in the Sen Sok precinct. All four of her children had been killed in the Khmer Rouge era. After liberation in 1979, though she had no children with her second husband, she had five step-grandsons and, developed powerful attachments with each. She said, “I loved each of them almost to the point of wanting to swallow them,” /srɑlaɲ stǝǝ leep/. She would /krɨɨt/ them every day and kiss them all over and down the belly. She would insert their penises into her mouth. Sometimes, if she bit one of them hard, he would cry out for his mother. When asked about the sexual connotation of her actions, she was startled to think that there could be such a thing.

### Family Members Versus the Community at Large

Most informants outside the immediate family said that if pain was not inflicted, it was normal to /krɨɨt/ a young boy. One father, VP, recollected that he had been hurt as a boy when some adults had pulled his genitals. VP was not alone in expressing concern that a boy could suffer physical damage this way, but not a single informant thought that this could happen within a family, but rather, only at hands of strangers who may be unwittingly overzealous or may harbor ill-will, but even then, none of the informants thought it could be sexual abuse.

### Signaling Benign or Malevolent Intentions

All these forms of /krɨɨt/ were considered “normal” and innocent rather than UST. Whenever the informants described making contact with a boy’s genitals, they bracketed the word with another, /leiŋ/, a serial verb that is commonly used by Cambodians in everyday language. The word /leiŋ/ has two senses: as a transitive, “I am playing” or “just for [my] fun,” and as a serial verb meaning that it was just a routine “normal” action. For example, a person reaches out and lightly scratches, /cʰək leiŋ/ (ឆឹកលេង); grabs, /cap leiŋ/ (ចាប់លេង); scratches and scrapes, /ckəh leiŋ/ (ឆ្កឹះលេង); and headbutts the boy’s upper body and comes in contact with his genitals, /ɲʊəl leiŋ/ (ញល់លេង) and, in so doing, collides with him, /cʊəl leiŋ/ (ជល់លេង). Another act seeks to get a reaction, like feigning a blow, /cʊənlɔɔ leiŋ/ (ជន្លលេង) and using one’s elbows and hands to grab the boy, /ɲʊəh leiŋ/ (ញោះលេង). The word /leiŋ/ signaled that these actions were non-sexual. Another way to signal that the action was in line with social norms was by adding the predicative /tʰoammeaʔtaa/ as an adverb to the attributive verb /leiŋ/. Most informants, even mothers such as KCC who had a personal experience of having been raped as a girl, maintained that she, like other villagers, believed that, no matter what the human rights organizations taught, genital touching of young boys, unlike that of girls, was /tʰoammeaʔtaa/. By adding /tʰoammeaʔtaa/ to /leiŋ/, even by duplicating the /tʰoammeaʔtaa/ more than once, they asserted that their actions were normal. When the informants depicted the action upon children as /bɑmpien/, ISL and US signified a violation, wittingly or unwittingly, in touching a boy. The notion of “grooming” a boy or girl for sexual purposes seemed foreign to our informants, and was understood in Khmer as /lbuǝŋ luǝŋ loom/ (ល្បួងលួងលោម). There is some ambiguity around whose intention matters—the toucher or the boy. It was understood that boys may not enjoy genital touching because it may hurt them physically, but no one considered whether the boys would feel distressed and abused. The view was that boys were too young to have sexual feelings and could therefore not be aroused. Sexual arousal, rather than trauma, was considered a marker of CSA.

## Discussion

The driver for genital touching by an adult is “attraction” toward the boy, a construction based on /krɨɨt/. Parents and caregivers who yield to their attraction to boys, either “/krɨɨt/” them all over their bodies, including their penises or feel drawn to the boys’ genitals. The relationship with the boy, in most cases is well-established and one of trust. There are positive motives, as the intention is to nurture and/or protect the boy and strengthen their bond. Posts on social media show how this works in popular imagination. There are numerous Facebook posts by parents who want to show their attraction to their adorable sons, with the parent saying, “You, my son, are so beautiful I want to eat you.” These posts show how they are led to /krɨɨt/ their sons, thus drawing the boys to feel reciprocally attached to the parent. In Cambodian society, which is hierarchical based on age, elderly people are credited as possessing three related qualities: social and cultural etiquette, /soʔciiveaʔtʰoa/ (តើសុជីវធម៌); morality, /sǝl tʰoa/ (សីលធម៌); and virtue and kindness, /kunaʔtʰoa/ (គុណធម៌). They are considered guardians of these qualities and as bearing the duty of care to guide the young. They are given some latitude to do so, while younger people are not. Children tend to gravitate toward their elders. Whether it is to express appreciation or to warn a child to cover their nakedness, an older person is a moral model and is not considered to have abusive intentions. If an elder touches a boy—not a girl—the parents tend to feel gratitude rather than suspicion. The traditional view was that boys are not sexually abused through genital touching, whereas girls can be. One can see how people decide whether the motive is well-intentioned or malevolent based on the sex of the child, as seen by their use and understanding of the compound verb /pah poal/ (ប៉ះពាល់).

### Anthropological Reflections

It may stretch credulity to say that touching the penis could be devoid of sexuality. The findings show that, at least for older people, reaching out to touch a male’s genitals—be it an adult or child—is driven by appreciation for the potency for reproduction, fertility, fecundity, and production of the seed, not only of the individual as a progenitor, but also of the fertility of the agrarian nation. In the archaeological roots of some Indianized states in South and Southeast Asia, including Cambodia, the *liṅga* is a stone phallic symbol, an emblem for the worship of Shiva. The ancient Vedic roots of the phallus are evident. In Khmer, the formal word for penis is /lɨŋ/ (លិង្គ), which has its origins in the Sanskrit word *liṅga*, and portrays masculine potency in which the penis is a symbol of power rather than of sex. This formal word is not used in the context of genital touching. Instead, in spoken Khmer, people call the penis and testicles using the compound word /pɔɔŋ kdɑɑ/ (ពងក្ដ), literally, “ovoid ball” and “dick.” This signals the mythical roots of continuity from father to son. In the word /kdɑɑ/ (ក្ដ), the nominative prefix /kɑɑ/, the first consonant of the Khmer alphabet, means “beginning to build,” and is added to the verb /tɑɑ/ (ត), meaning “to continue.” Figuratively, the phallus signifies fertility, agricultural productivity, survival, and continuity. It is possible that touching a boy’s penis unconsciously carries this metaphor.

The findings reveal a few negative motives for genital touching by adults. This is not to say that people were unaware of the possibility that touching a boy’s genitals could lead to accusations. There is a Khmer saying, “If you play with children, you will cloud your reputation, if you play with a serpent, you will get bitten,” /leiŋ kmeɛŋ? apyʊəh, leiŋ pʊəh pʊəh kʰam/ (លេងក្មេងអាប់យស លេងពស់ៗខាំ), and a linked Buddhist saying that the moment of being bitten by a snake does not seem harmful, but the problem manifests later. “Playing with children” may be a euphemism for inappropriate sexual behavior toward a boy. Conveying one’s innocent intention is important, never more so than in contemporary society where village heads and authorities are on the lookout for CSA, and the family man “playing” with his son would never have imagined that he may be “bitten,” that is, arrested for abusing his beloved son. Even if a parent or caregiver touches the boy’s genitals with the purest of motives, the act could still be experienced by the child as unwanted, a fact that could easily escape the notice of the adult.

The action can also show semantic roots of sexuality. In /cʰək/, the person intentionally reaches out to touch another without a sexual connotation. The word is adapted from the onomatopoeic repetitive reduplicative, /cʰək-cʰək/ (ឆឹកៗ), as in the sound of two stones being rubbed together to make fire. Thus, there is an allusion to the sexual arousal that is kindled by physical stimulation. Metaphorically, /cʰək-cʰək/ implies the kindling of sexual passion through foreplay.

The significance of genital touching varies by age and developmental stage of the boy. It is common for a parent or caregiver to fondle the genitals of an infant or toddler, and for this to continue throughout childhood, although it would be unusual for it to continue after puberty. Boys touching or grabbing one another can also extend through childhood, even into adolescence.

The metaphors and symbolic language of genital touching expressed by adults are intriguing. Some used Rahu as a metaphor for a parent who is overcome by desire to devour their child, for example, by touching his genitals. There is an awareness that acting without a limit can destroy the target of the parent’s desire. Parents cannot smother their babies in an overwhelming embrace, and an impassioned lover cannot risk killing their sexual partner. Restraint is important. The metaphor of the maturing boy as a fish symbolizes his virility, as his penis has grown to maturity. We also found metaphors in which child development is represented in the world of livestock. Some informants applied the imagery of calves growing into full-grown cattle to that of an unclothed young boy in the village with his yet-immature genitals on display.

In the spectrum of action, contact with the body is richly nuanced and can involve any part of a person’s body, such as the arms, head, or back, and may even involve an embrace of an infant or child. The semantics are informed by and inform the culture in turn. The compendium of Khmer action verbs can be taken by a western observer as an indicator of both physical abuse and rough play beyond what is normal. It is misleading, however, to interpret these verbs as they stand because action words in Khmer, unlike those in English, should be interpreted by considering how they are employed as part of serial verbs. Word modifiers signal that the action is not as malevolent as may first be thought. In Khmer, /leiŋ/ stands on its own as a transitive verb. The lexical meaning in English is “playing.” However, the word is also used as an attributive verb /leiŋ/, meaning that what was said must not be taken seriously. In this case, the person uses /leiŋ/ as though to cancel out the initial verb he used it with. The scene is set for a mistranslation, as in, “I really did it, it was just for fun, that is, for my own amusement at his expense.” Instead of hearing the man’s words as he intended them, they tend to be heard as an admission of guilt and indifference to the impact of the adult’s conduct on the boy. If a Cambodian says, “I touched his penis” + the word /leiŋ/, in English, his statement could wrongly be taken to mean that he had sexual intentions. The Khmer grammatical structure tells another story. Adults described their conduct using the above Khmer serial verbs, in which the Khmer verb /leiŋ/ is a trailing constituent in the post-position, which functions as an “attributive verb” that the action is simply in jest. Cambodians interpret /tʰoammeaʔtaa/ to mean that something is literally “natural.” This idea of normalcy comes through in rural life, which is filled with work from dawn to dusk, where one’s hands are always busy plowing, as captured by the Khmer proverb, “busy hands make for a full stomach,” /rɔpɨh day ptey cʔaet/ (រពឹសដៃផ្ទៃឆ្អែត). In rural Cambodia, the same sense of normalcy is conveyed about the genital touching of boys.

### The Cultural Borderland Between Normality and CSA

The findings raise intriguing questions on the borderland between love and abuse and, beyond that, to a reconsideration of what is commonly designated as harmful traditional practices (Campbell, 2020) and harmful cultural practices (Longman et al., 2016). Dauth and Ruggiu (2020) addressed the “homage to the penis,” an expression coined by Money et al. (1991) as being practiced worldwide. Money et al. (1991) described a Telugu-speaking group in Andhra Pradesh in India, where, after a boy turns one, the father and other adult male kin “bounce a kiss of approval off his penis” and over succeeding years, “flick or pull the foreskin . . . and throw the kiss back to the penis” (p. 2). Dauth and Ruggiu (2020) argued that “homage to the penis” of the child has been practiced throughout much of European history. Josephs (2011) reviewed the primal scene in cross-cultural and psychoanalytic perspectives and argued that genital fondling has been considered normal even in certain western contexts. It may well be “normal” but may sometimes still be abusive. There is the celebrated example cited by Fishman (1982, p. 272), where the ladies of court took the future Louis XIII (1601–1643), King of France and Navarre, as a child aged 2 years, to bed with them to waggle his “cheater.” For Ariès (1962), this was normal practice at the time because there was no concept of childhood and therefore the boy was indifferent to sex, a view strongly opposed by DeMause (1995). Even if men in Cambodia are satisfied that genital touching of boys is a positive expression of love, they may find themselves standing accused of sexual abuse in western jurisdictions.

### A Final Caution

Genital touching of young boys in Cambodia remains accepted as normal, at least in rural settings, but that does not make it “defensible” or “risk free.” The findings touch upon cultural relativism and traditional practices involving the penis; for example, Robinson and Hammer (2014) described the Haredi Jewish practice of *metzitzah b’peh* in which the circumcising rabbi sucks the bleeding penis. White (1999) cautioned against the misuse of cultural insights to justify abuse. The findings on /kriit/ could be useful, in White’s terms, as a “principle of respect” for the ways of life of Cambodians, a “tool of learning and understanding” of the emic expressions of affection and care for a child in Khmer society, a “useful corrective to pseudo-universalist notions” of UST, but in so doing, it should not be “a basis for legitimizing whatever we may see.”

Even though none of the informants had heard of the Law on Suppression of Human Trafficking and Sexual Exploitation (2008), they were aware that sexual conduct of all kinds with a minor is illegal, and were conscious that the age of consent was 15 years. Interpreting the phrase “in a sexual way” poses a challenge because of a certain cultural bashfulness about discussing sexual matters at all. Further, Vijghen et al. (2014) “Both adults and children understood child sexual abuse narrowly as the penetrative rape of girls . . . [and] Indeed, sexual abuse was not widely perceived as something that could happen to boys,” (p. 9), which is consistent with our experience. If it can be shown that in Cambodia, it is “normal” to touch a boy’s genitals to express affection or for any other reason that is not “in a sexual way,” *and that there are no apparent adverse consequences for the boy*, is it necessarily CSA? A lingering question is whether genital touching, even if motivated by care and love rather than for sexual gratification, may have adverse consequences for the boy.

### Limitations

The fact that this study gathered data from adult informants and not from boys, is a limitation. Older boys do not always want their genitals touched. The report on Cambodia’s Violence Against Children Survey (Ministry of Women’s Affairs et al., 2014) described two adverse effects. “Young men aged 18 to 24 years admitted that they did the same thing to younger boys now that they were older and no longer in fear of it happening to them. They said that they had learned to copy the behaviors of older men around them” (p. 136). Second, many boys said they had been teased by adults who pulled their trousers or shorts down and exposed their genitals. They reported being tormented by adults who both threatened to cut off their penises and tugged on their genitals. The boys felt fearful, disempowered, humiliated, enraged, and helpless. “They could not explain to adults how it made them feel. This highlights the difference in perceptions of actions by adults and children. What the adults viewed as harmless, the children viewed as harmful” (p. 136). The age of a young person will also affect their perception of UST (Shepherd, 2022). It is essential to document, wherever possible, the experiences of the boys and what they mean by “unwanted” sexual contact, as various researchers, such as Bagley (1990), have done. Another limitation is that the study is limited to the perspectives of adults from the traditional collectivistic rural social milieu. The changing cultural norms in modern urban Cambodia complicate the analysis. Research must focus on clashes between the law and such cultural practices, and identify whether there have been judicial precedents or legal commentaries on this issue.

## Conclusion

The concept of /krɨɨt/, so natural to rural Cambodians, has never been reported in the English-language literature and remains unknown in the world of child protection. The genital touching of boys, driven by /krɨɨt/, is not generally seen by rural Cambodians as harmful. If anything, it is considered beneficial in forging emotional bonds. Effective education programs on CSA prevention are more likely to be successful if, rather than applying an “outside-in” approach of conveying the universal rights of the child, they employ an “inside-out” view where the Cambodian *emic* concepts such as /krɨɨt/ are taken as the starting point. If child protection authorities are to engage successfully with rural communities to promote the rights and safety of boys, they must be informed of the cultural meaning of /krɨɨt/. In further work, reported separately, we explored the perceptions of various stakeholders such as ordinary villagers, village heads, and Buddhist monks and ritual officiants toward the educational workshops provided by rights-based organizations. Their observations on genital touching are hardly remarkable to Cambodians and those who have had the opportunity to share life with the Khmer and observe their child rearing practices. However, the documentation of /krɨɨt/ now enables an evidence-based conversation. Is the action of /krɨɨt/ to be considered a “harmful traditional practice” to be educated out of existence? An essentialist inward-out cultural lens can be seen as a justification for abuse, whereas using an outward-in lens calling for the outlawing of “harmful traditional practices” could be seen as introducing other complications in its wake. From the community perspective, probably not, if the exceptional cases with sexual or malicious intent are weeded out. From the children’s perspective, however, more research is needed to understand the impact of this practice.

## Notes

1. We extended our focus to include the genital touching of boys, and in this study, with its aim of describing the meaning of behavior that is normative for ordinary rural people from traditional collectivistic communities, I excluded temple boys and novice monks and boys who are victims of trafficking and sex work. Data related to sexual labor and trafficking have also been excluded.

2. The escalation is shown in the manner in which the informants used the action verb in word formation. The basic verb /cʰək/ (ឆឹក), depicts a benign stage of rubbing. In the next level of severity, the informants depict the action using the entire reduplicative /cʰək cʰək/ (ឆឹកៗ), that is, repeated rubbing. The level of severity is increased by the prefixation [prɑ-], [ប្រ-] to make /prɑcʰək/ (ប្រឆឹក), meaning that the rubbing is above and beyond the baseline of /cʰək/. In a related representation of the escalated severity of the action of /cʰək/, the informants add a causative prefix [bɑɲ-], [បញ់-] when they say that the action is /bɑɲcʰək/ (បញ្ឆឹក), as if the person wants to stimulate the boy no less than to obtain gratification for themselves. The meaning comes as a part of the derivative term.
